# Directed Evolution Pipeline for the Improvement of Orthogonal Translation Machinery for Genetic Code Expansion at Sense Codons

**DOI:** 10.3389/fchem.2022.815788

**Published:** 2022-02-17

**Authors:** Wil Biddle, David G. Schwark, Margaret A. Schmitt, John D. Fisk

**Affiliations:** Department of Chemistry, University of Colorado Denver, Denver, CO, United States

**Keywords:** genetic code expansion, sense codon reassignment, noncanonical amino acid, synthetic biology, directed evolution, protein engineering

## Abstract

The expansion of the genetic code beyond a single type of noncanonical amino acid (ncAA) is hindered by inefficient machinery for reassigning the meaning of sense codons. A major obstacle to using directed evolution to improve the efficiency of sense codon reassignment is that fractional sense codon reassignments lead to heterogeneous mixtures of full-length proteins with either a ncAA or a natural amino acid incorporated in response to the targeted codon. In stop codon suppression systems, missed incorporations lead to truncated proteins; improvements in activity may be inferred from increased protein yields or the production of downstream reporters. In sense codon reassignment, the heterogeneous proteins produced greatly complicate the development of screens for variants of the orthogonal machinery with improved activity. We describe the use of a previously-reported fluorescence-based screen for sense codon reassignment as the first step in a directed evolution workflow to improve the incorporation of a ncAA in response to the Arg AGG sense codon. We first screened a library with diversity introduced into both the orthogonal *Methanocaldococcus jannaschii* tyrosyl tRNA anticodon loop and the cognate aminoacyl tRNA synthetase (aaRS) anticodon binding domain for variants that improved incorporation of tyrosine in response to the AGG codon. The most efficient variants produced fluorescent proteins at levels indistinguishable from the *E. coli* translation machinery decoding tyrosine codons. Mutations to the *M. jannaschii* aaRS that were found to improve tyrosine incorporation were transplanted onto a *M. jannaschii* aaRS evolved for the incorporation of *para*-azidophenylalanine. Improved ncAA incorporation was evident using fluorescence- and mass-based reporters. The described workflow is generalizable and should enable the rapid tailoring of orthogonal machinery capable of activating diverse ncAAs to any sense codon target. We evaluated the selection based improvements of the orthogonal pair in a host genomically engineered for reduced target codon competition. Using this particular system for evaluation of arginine AGG codon reassignment, however, *E. coli* strains with genomes engineered to remove competing tRNAs did not outperform a standard laboratory *E. coli* strain in sense codon reassignment.

## Introduction

**FIGURE 1:**
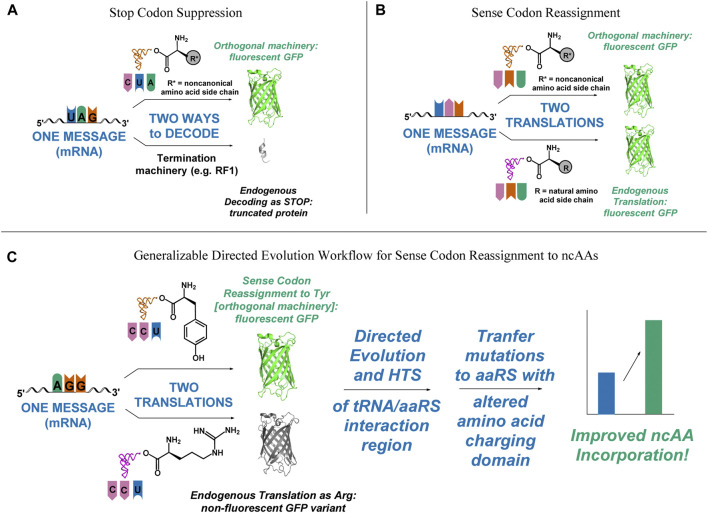
Visual representation of outcomes of successful and unsuccessful selection schemes, depicting the need for a fast, functional screen for improving sense codon reassignment. **(A)** In stop codon suppression, only successful incorporation of the ncAA leads to functional full-length protein. Missed incorporations lead to truncated products. **(B)** In sense codon reassignment, incorporation of either the desired ncAA or a canonical amino acid lead to full-length, functional protein. **(C)** A directed evolution pathway that incorporates improvement of sense codon reassignment using a readily screenable reporter, followed by transplantation of the mutations that gave rise to the improved reassignment efficiency onto an orthogonal pair that incorporates a ncAA would lower one of the major barriers to utilizing sense codon reassignment for expansion of the genetic code to 22 amino acids and beyond.

The genetically-encoded introduction of noncanonical amino acids (ncAAs) is a powerful tool for increasing the chemical diversity of proteins because it enables the precise placement of desired side chain functionality within a growing peptide chain. Two general approaches to expand the genetic code have been widely employed: nonsense suppression and residue specific reassignment (Figure 1) (Wiltschi and Budisa, 2007; Ngo and Tirrell, 2011). Advances in both technologies have increased the efficiency with which a single ncAA can be genetically encoded. Nonsense suppression technology has been improved through genome modifications, enabling the elimination of the release factor that typically competes to read the amber stop codon as a termination signal (Mukai et al., 2010; Johnson et al., 2011; Lajoie et al., 2013). Amino acid-specific reassignment has been updated by breaking the degeneracy of the genetic code to enable the reassignment of individual sense codons (Kwon et al., 2003; Bohlke and Budisa, 2014; Zeng et al., 2014; Lee et al., 2015; Mukai et al., 2015; Ho et al., 2016; Kwon and Choi, 2016; Wang and Tsao, 2016). Improvements in both methods have focused primarily on genetic additions and deletions rather than on functional improvements of the evolved orthogonal translation components central to both technologies.

*In vivo* incorporation of ncAAs by either nonsense suppression or sense codon reassignment (SCR) requires the introduction of an orthogonal aminoacyl tRNA synthetase (aaRS) engineered to recognize and attach a ncAA onto its cognate, orthogonal tRNA (Furter, 1998; Wang et al., 2001). The orthogonal tRNA is not recognized by the set of endogenous aaRSs, and the orthogonal aaRS does not recognize the complement of endogenous tRNAs. The vast majority of the ribosomally incorporated ncAAs have been introduced using derivatives of only two orthogonal pairs: the tyrosine tRNA/aaRS pair from *Methanocaldococcus jannaschii* (*M. jannaschii*) and the pyrrolysine tRNA/aaRS pair from *Methanosarcina* species. (Wang et al., 2001; Polycarpo et al., 2006). Variants of these pairs that recognize and aminoacylate over 150 different ncAAs have been developed (Liu and Schultz, 2010; Wan et al., 2014; Dumas et al., 2015). Continuing efforts to find or engineer new orthogonal pairs have identified several additional potential orthogonal systems. These systems, however, have not yet found widespread use in genetic code expansion (Cervettini et al., 2020; Ding et al., 2020; Zhao et al., 2021).

Here, our previously-developed fluorescence-based screen is utilized as the first step in a directed evolution workflow to improve the incorporation of a ncAA in response to the Arg AGG sense codon (Kuhn et al., 2010; Biddle et al., 2015). The fluorescence-based screen takes advantage of the absolute requirement of tyrosine in the central position of a Thr-Tyr-Gly triad for mature GFP fluorophore formation. We first screened a library with diversity introduced into both the orthogonal *M. jannaschii* tyrosyl tRNA anticodon loop and the cognate aaRS anticodon binding domain for variants that improved incorporation of tyrosine in response to the AGG codon. The most efficient variants produced fluorescent protein at levels indistinguishable from the *Escherichia coli* (*E. coli*) translation machinery decoding Tyr codons, and reassignment efficiency improved from 56.9 ± 2.4% to 98.6 ± 4.7%. Mutations to the *M. jannaschii* aaRS that were found to improve tyrosine incorporation were transplanted onto a *M. jannaschii* aaRS evolved for the incorporation of *para*-azidophenylalanine (pAzF) (Chin et al., 2002). The same suite of mutations improved incorporation of pAzF in response to the AGG codon from 29.5 ± 0.1% to 50.1 ± 1.1% as measured *via* fluorescence, an approximately 1.7 fold improvement for both amino acids. The improved *para*-azidophenylalanine incorporating tRNA/aaRS pair was evaluated in strains from which competition for the AGG codon was reduced by deletion of the gene for the primary endogenous tRNA that reads the AGG codon (Lee et al., 2015). Improvements in sense codon reassignment efficiency due to reduced competition did not appear to combine with improvements selected through directed evolution. We have previously quantified the reassignment efficiency of several sense codons by both the natural, tyrosine-incorporating *M. jannaschii* and an evolved tyrosine-incorporating *M. barkeri* orthogonal pair (Schmitt et al., 2018; Schwark et al., 2020; Schwark et al., 2021). This workflow should be generalizable for improving incorporation of other ncAAs in response to other sense codon targets.

Nonsense (stop codon) suppression has been the most widely used method for introduction of noncanonical amino acids into the genetic code. In *E. coli*, the amber stop codon is employed both because it is a termination signal and because it is the least frequently used codon. Nonsense suppression utilizes orthogonal aaRS variants to aminoacylate >150 different ncAAs onto essentially an identical cognate tRNA with a CUA anticodon. Variation across orthogonal systems that incorporate different ncAAs primarily resides within the amino acid recognition domain of the aaRS. tRNA/aaRS recognition is typically not altered across variants. The kinetic efficiencies of aaRSs evolved to recognize ncAAs are generally poor. *In vitro* kinetic aminoacylation efficiencies for these enzymes are on the order of 1% that of natural enzymes (Guo et al., 2014; Amiram et al., 2015; Rauch et al., 2016). The measured efficiency of ncAA incorporation is dependent on systemic variables beyond the kinetics of aminoacylation (e.g. ncAA, tRNA and aaRS concentrations), and single site incorporation efficiencies of 10–30% are readily achievable. For systems in which a single amber stop codon is targeted for ncAA incorporation, this strategy and enzyme function level are sufficient to produce modified proteins in usable quantities. The expected protein yield drops as the *n*th power of the single site incorporation efficiency (where n is the number of incorporations attempted). Even for a highly efficient (e.g. 50%) ncAA-incorporating tRNA/aaRS system, the expected protein yield for a protein containing 3 amber stop codons would be 12.5% of the yield expected if the amber codon were a typical canonical amino acid (Schwark et al., 2018).

Advances in the technology that underpins both amber stop codon suppression and sense codon reassignment by breaking the degeneracy of the genetic code have focused primarily on extrinsic system improvements, such as increasing the expression level of the orthogonal pair, improving the interactions of the orthogonal tRNA with the endogenous translation components (e.g. EF-Tu), or reducing endogenous competition for the targeted codon. The orthogonal translation components are orthogonal because they are taken from a phylogenetically distant species, and the efficiency of incorporation could potentially be increased by improved assimilation of the orthogonal components into the host organism. A large amount of “orthogonal space” in the vicinity of each orthogonal system appears to exist, as mutually orthogonal variants are readily evolvable (Neumann et al., 2010; Willis and Chin, 2018; Cervettini et al., 2020). Orthogonal machinery levels have been adjusted by expressing both tRNAs and aaRSs from cassettes with different promoters in plasmids with different copy numbers (Ryu and Schultz, 2006; Young et al., 2010; Chatterjee et al., 2013). A handful of attempts to improve amber stop codon suppression *via* tRNA mutation have focused on changes to tRNA sequences that modulate interactions with elongation factor Tu (EF-Tu) (Guo et al., 2009; Schrader et al., 2011; Mittelstaet et al., 2013; Fan et al., 2015; Maranhao and Ellington, 2017). The extent to which tRNA mutations that improved incorporation of one ncAA in response to amber codons functioned when transferred between aaRSs specific for different ncAAs suggested that the extent of transfer is idiosyncratic, although in general, improved variants were identified (Guo et al., 2009). Reducing the competition from endogenous translation components has been the major mode of improving genetic code expansion systems. Three separate approaches involving different combinations of organismal genomic modifications enabled the deletion of the gene for the release factor that typically reads the amber stop codon as a termination signal (Mukai et al., 2010; Johnson et al., 2011; Lajoie et al., 2013). Additional genome rewriting projects seek to generate multiple “free” codons for ncAA incorporation (Ostrov et al., 2016; Wang et al., 2016; Fredens et al., 2019). Attempts to improve sense codon reassignment have involved deletion of competing tRNA genes and antisense RNAs to reduce competition between orthogonal and host components reading the AGG arginine codon (Zeng et al., 2014; Lee et al., 2015).

An alternative strategy for improving the efficiency of orthogonal tRNA/aaRS pairs targets interactions intrinsic to the cognate pair itself. The anticodon is often an important identity element that allows a specific aaRS to recognize its appropriate tRNA (Giege et al., 1998). Changing the anticodon of the *M. jannaschii* tRNA, even to CUA for amber suppression, is known to strongly affect the efficiency of aminoacylation (Fechter et al., 2001). Increasing the recognition between the orthogonal tRNA and the anticodon binding domain of the aaRS is expected to lead to a higher concentration of aminoacylated tRNA and better kinetic competition against endogenous tRNAs capable of decoding the targeted codon. Improvements of the interactions between the components of the orthogonal translation machinery should facilitate improved incorporation efficiency with less dependence upon the system characteristics into which orthogonal translation system is placed. Several general selection strategies have been developed to improve the function of stop codon suppressing orthogonal pair systems, although the maturation of initially selected aaRSs utilized for ncAA incorporation is not commonly performed (Pott et al., 2014; Wang et al., 2015; Rauch et al., 2016; Maranhao and Ellington, 2017; Owens et al., 2017). Directed evolution of *M. jannaschii* tRNA/aaRS pair variants for improved reassignment of the amber stop codon as well as Lys AAG and His CAU sense codons have been described (Takimoto et al., 2009; Kuhn et al., 2010; Mittelstaet et al., 2013; Biddle et al., 2015; Wang et al., 2015; Biddle et al., 2016).

A major obstacle to using directed evolution to improve the efficiency of sense codon reassignment is that fractional sense codon reassignments lead to heterogeneous mixtures of full-length proteins with either a ncAA or a natural amino acid incorporated in response to the targeted codon. In stop codon suppression systems, missed incorporations lead to truncated proteins and improvements in activity can be inferred from increased protein yields or the production of downstream reporters (Figure 1A). In sense codon reassignment, heterogeneous mixtures of full-length proteins greatly complicate the development of screens for variants with improved activity (Figure 1B). Implementation of a general workflow that allows selection of orthogonal pair variants that reassign a particular sense codon first by evaluating variants with a fast, facile screen (e.g. fluorescence-activated cell sorting, FACS) followed by subsequent transfer of the mutations to a version of that orthogonal pair engineered to recognize a ncAA, for which screening would be more challenging, would lower the barrier to wider utilization of sense codon reassignment for genetic code expansion (Figure 1C).

## Methods and Materials

The Supplementary Materials file includes general reagents and materials, as well as detailed experimental protocols for site-directed mutagenesis, the fluorescence-based screen, and protein expression and isolation for reassignment efficiency analysis and mass spectrometry (Kunkel, 1985; Gao et al., 2003; Clackson and Lowman, 2004). This file also includes a graphical representation of the directed evolution workflow (Supplementary Figure S1), an image of a representative protein gel (Supplementary Figure S2), and a representative optical density *vs.* time plot for reassigning systems (Supplementary Figure S3). Finally, further experimental information, including a list of the oligonucleotide primers used for library creation, cell strain information, and preparation of electrocompetent cells are provided (Sambrook and Russell, 2001).

## Results and Discussion

### Co-evolution of the *M. jannaschii* tRNA_CCU_/TyrRS Improves Reassignment of AGG Codons to Tyrosine

We selected the arginine AGG codon for initial evaluation of this directed evolution workflow because AGG has been the most common target for sense codon reassignment using variants of both the orthogonal pyrrolysyl and *M. jannaschii* tyrosyl tRNA/aaRS pairs (Krishnakumar et al., 2013; Zeng et al., 2014; Lee et al., 2015; Mukai et al., 2015; Wang and Tsao, 2016). Zeng et al. reported approximately 90% efficient reassignment using a variant of the *Methanosarcina* pyrrolysyl tRNA/aaRS pair and media in which the concentration of arginine was controlled (Zeng et al., 2014). Mukai et al. mutated the 38 AGG codons found in essential *E. coli* genes to other arginine codons and subsequently removed the gene for endogenous tRNA_CCU_, *argW*, from the genome (Mukai et al., 2015). These genetic transformations allowed incorporation of a close structural analogue of arginine at very high levels using a variant of the *Methanosarcina* pyrrolysyl tRNA/aaRS pair. Lee et al. described reassignment of the AGG codon using a variant of the *M. jannaschii* tyrosyl tRNA/aaRS pair (Lee et al., 2015). Their strategy also utilized an *argW* knockout strain, however the distribution of AGG codons in the genome was not adjusted. The fact that *argW* knockout strains are viable and exhibit only slightly reduced growth suggests that the remaining *E. coli* tRNA_UCU_ is able to read AGG codons to some extent *via* an expected G/U wobble interaction. Double knockouts of both *E. coli* tRNA_UCU_ and tRNA_CCU_ are not viable (Mukai et al., 2015).

The efficiency of AGG reassignment was quantified in *E. coli* DH10B expressing the *M. jannaschii* tyrosyl aaRS, its cognate tRNA^Opt^ with a CCU anticodon (*M. jannaschii* tRNA_CCU_/TyrRS), and a GFP reporter protein with an arginine AGG codon specifying the central fluorophore position (Young et al., 2010; Chatterjee et al., 2013; Biddle et al., 2015). The gene sequence of the GFP reporter uses a reduced codon set such that instances of the 20 sense codons decoded through wobble interactions and 10 additional least frequently used sense codons are eliminated or greatly reduced from the gene (Schmitt et al., 2018). No AGG codons are present in the aaRS gene. Changing the anticodon of the *M. jannaschii* tRNA^Opt^ to Watson—Crick base pair with the AGG codon results in 56.9 ± 2.4% reassignment efficiency. Significantly, this high efficiency is evident in rich media without any modifications to the *E. coli* genome or alterations to the orthogonal tRNA and aaRS beyond the anticodon change. Reassignment efficiency is quantified by normalizing the observed fluorescence with a test codon at the fluorophore position to a “100% fluorescence” reference value determined by expressing sfGFP with a tyrosine UAC codon at position 66. A “0% fluorescence” reference is determined by expressing sfGFP with a non-tyrosine codon specifying the fluorophore. Both fluorescence reference systems include a plasmid expressing the orthogonal machinery to maintain a similar metabolic burden on each system.

**FIGURE 2:**
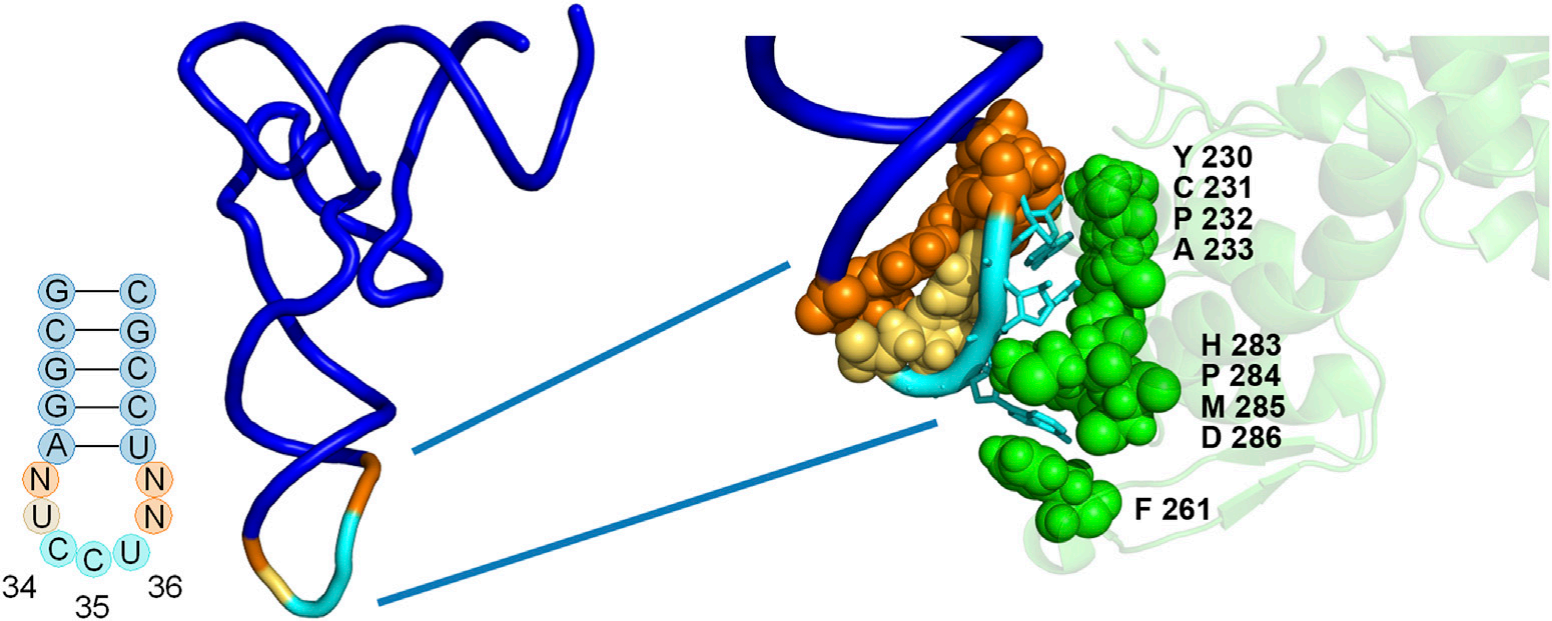
Positions of library mutations mapped onto the co-crystal structure of the *M. jannaschii* tRNA/aaRS pair (PDB 1J1U). Positions 32, 37, and 38 within the tRNA anticodon loop were allowed to vary in conjunction with 9 amino acids in the anticodon binding domain of the aaRS. The diversity available at each position in the aaRS is described both in the text and in Table 1.

A library of *M. jannaschii* tRNA anticodon loop and aaRS anticodon binding domain variants was constructed and screened for improved efficiency of reassignment of the AGG codon to Tyr (Figure 2). Co-evolution of the tRNA anticodon loop and the aaRS anticodon binding domain was hypothesized to increase the recognition and aminoacylation of the tRNA by the aaRS, which should, in turn, increase the effective concentration of the aminoacylated tRNA and lead to better competition against endogenous tRNAs for decoding the targeted codon. Furthermore, the positions within the *M. jannaschii* Tyr aaRS that contact the cognate tRNA anticodon are spatially distant from and within a separate domain relative to the amino acid binding pocket, suggesting that mutations which lead to improvement of incorporation for one amino acid may be transferable to a variant of the same enzyme that recognizes and aminoacylates a different amino acid (Kobayashi et al., 2003).

The library included diversity at 9 amino acid positions in the aaRS and 3 of 4 nucleotides in the tRNA anticodon loop outside of the anticodon (32, 37, and 38) (Biddle et al., 2015). The varied amino acid positions were chosen based on the proximity to the nucleotides of the anticodon in the co-crystal structure of the *M. jannaschii* tyrosyl tRNA/aaRS orthogonal pair (PDB 1J1U) (Figure 2) (Kobayashi et al., 2003). Amino acid residues with side chains within 5Å of anticodon nucleobases were varied. Amino acid diversity at each of the positions was limited in order to maintain a manageable library size, and degenerate nucleotides were selected to include the parent *M. jannaschii* aaRS amino acid at each position (Table 1). Amino acids 230-233 were allowed to vary between four amino acids. Greater diversity was included at the remaining 5 positions; diversity at positions 261 and 283-286 varied between 9 and 15 different amino acids. Three of the four nucleotides flanking the anticodon in the tRNA were allowed to vary; the universally conserved U33 was preserved (Figure 2; Table 1). The library had a theoretical diversity of 4.1 × 10^10^ at the DNA level, 4.1 × 10^9^ at the expressed level.

**TABLE 1:** Sequences and AGG reassignment efficiencies for *M. jannaschii* tyrosyl tRNA/aaRS variants selected from the library.

	Amino acid positions in aaRS anticodon binding domain									Nucleotide positions in tRNA					AGG % Eff^a^
	230	231	232	233	261	283	284	285	286	32	33	CCU	37	38	
*M. jannaschii* tRNA/aaRS	Y	C	P	A	F	H	P	M	D	C	U		A	A	56.9 ± 2.4%
—	amino acid residue diversity									nucleotide diversity					—
—	4	4	4	4	15	13	12	9	12	4	1	—	4	4	—
—	degenerate nucleotide triplet for each codon									degenerate nucleotide					—
—	KMT	TNT	MMG	KYC	NHH	VDS	BNT	WBB	VVW	N	U	—	N	N	—
C3^b^	Y	F	Q	A	F	L	R	S	G	A	U	—	A	G	98.6 ± 4.7%
F3^b^	S	F	T	A	F	L	R	S	A	A	U	—	A	G	86.6 ± 3.0%
F8	D	F	T	A	F	L	R	T	A	A	U	—	A	G	88.2 ± 1.5%
H1 (2)^c^	A	Y	T	A	F	L	R	S	A	A	U	—	A	G	99.2 ± 2.3%
D1	D	Y	T	A	Y	L	R	S	H	A	U	—	A	G	94.4 ± 2.6%
F6 (2)^c^	S	Y	K	A	Y	L	R	S	N	A	U	—	A	G	95.5 ± 1.1%
D6	A	Y	Q	A	F	Y	R	S	H	A	U	—	A	G	83.2 ± 0.9%
G4^b^	D	C	K	F	I	R	S	W	H	G	U	—	A	G	98.6 ± 2.1%

a Error bars on reassignment efficiencies are the standard deviation of 12 biological replicates of each system, with the exception of *M. jannaschii* tRNA_CCU_/TyrRS, and tRNA_CCU_-C3/TyrRS-C3, which comprise evaluation of 24 biological replicates.

b Three clones (C3, F3, and G4) included mutations within the aaRS, outside of the varied positions. Clone C3 has E221K and R223G. Clones F3 and G4 have K228N.

c The number in parenthesis indicates the number of clones of the 10 characterized that had the listed sequence.

The constructed library contained 3 × 10^8^ unique transformants, which were allowed to multiply 25-fold (∼10^9^ cells). Of these, approximately 10^7^ cells were screened using fluorescence activated cell sorting (FACS), and the brightest 2% of cells were collected. Following amplification, the population was resorted, and the top 1% of fluorescent cells were collected. After amplification and a third round FACS, a portion of the collected cells were plated for single clone analysis (Supplementary Figure S1 in Supplementary Material). 75 of 84 visibly green clones showed increased fluorescence relative to the starting *M. jannaschii* tRNA_CCU_/TyrRS. The clones displayed a range of reassignment efficiencies, with the vast majority reassigning the AGG codon at greater than 75% efficiency. 10 of the 84 clones were selected for further analysis. Sequencing revealed eight unique sets of *M. jannaschii* tRNA_CCU_/TyrRS variants (Table 1). Because false positives resulting from reporter fluorophore revertants or mutations increasing the expression of GFP have been observed in other aaRS selections, the orthogonal translation machinery plasmids were isolated and retransformed into cells with unselected GFP reporter plasmids. Analysis of the eight identified *M. jannaschii* tRNA_CCU_/TyrRS variants suggested that the mutations within the tRNA and aaRS were responsible for the improved incorporation of tyrosine in response to the AGG codon. Sense codon reassignment efficiencies ranged from 83.2 ± 0.9% to 99.2 ± 2.3% (measurements from twelve colonies of each variant).

Strong consensus was apparent across the sequences of the selected *M. jannaschii* tRNA_CCU_/TyrRS variants (Table 1). In the case of the tRNA anticodon loop, the nucleotides identified at the three varied positions were identical for seven of the eight aaRS variants. The predominantly selected tRNA sequence was 5′-auCCUag-3′, where loop positions are written in lower case and anticodon nucleotides in upper case. Positions 32 and 38 were different than the starting *M. jannaschii* tRNA_CCU_: 5′-cuCCUaa-3’. While the predominant tRNA variant included purine nucleotides at the 3 varied positions, the majority of *E. coli* tRNAs include a pyrimidine at position 32 and a purine at 38 as in the starting sequence. No *E. coli* tRNA has a purine in both positions 32 and 38. A purine/pyrimidine relationship also exists for tRNA positions 33/37, with the universally-conserved uridine at position 33. In *E. coli*, the choice of purine at position 37 is strongly dependent on the nucleotide at position 36. The majority of *E. coli* tRNAs feature adenosine (or a modified adenosine) at position 37; only a few of the tRNAs with G36 anticodons have a modified guanosine at position 37 (Machnicka et al., 2013).

The seven aaRS sequences (from nine characterized variants) that were selected along with the single tRNA sequence have strikingly similar amino acid sequences (Table 1). Alanine, the amino acid present at position 233 in the original aaRS, was present in each of the seven sequences. Despite having the greatest opportunity to vary, position 261 maintained a similar amino acid to the wild type aaRS, with all seven sequences either remaining Phe or mutating to Tyr. The changes selected in the remaining seven varied positions modified the amino acid size and polarity relative to the amino acid present in the starting aaRS. In all seven selected sequences, position 284 mutated from Pro to Arg. Six of seven sequences included H283L and M285S. For both Leu 283 and Ser 285, the multiple codons for each amino acid available as a result of degenerate codon choice were present at the DNA level. All seven sequences included an aromatic residue at position 231. Less of a consensus was apparent at positions 232 and 286. Position 232 universally changed from the proline found in the starting aaRS with a weak consensus for Thr (4/7 sequences). Position 286 showed a weak consensus for alanine (3/7 sequences) or histidine (2/7 sequences). The amino acids identified at position 230 were evenly distributed between Tyr, Ala, Asp, and Ser, the 4 amino acids available at that position. The single selected tRNA with a different anticodon loop sequence (5′-guCCUag-3′) was co-selected with an aaRS with a unique suite of mutations (clone G4 tRNA/aaRS pair). The amino acids selected at six of nine varied positions in clone G4 were not identified in any of the other variants.

Three of the ten clones, including the highly efficient co-evolved tRNA/aaRS pair C3, had additional, spontaneous mutations to the aaRS sequence. Clone C3 had two mutations: E221K and R223G. Clones F3 and G4 had a K228N mutation. All three of these mutations occur within an *α*-helix near the anticodon binding domain. The E221K and R223G mutations in C3 occur near the end of the *α*-helix furthest removed from the tRNA. The K228N mutation was also observed in three of ten characterized clones identified from a similar tRNA anticodon loop/aaRS anticodon binding domain library targeting improvement of Lys AAG codon reassignment (Biddle et al., 2015). The anticodons required to Watson—Crick base pair with Lys AAG (CUU) and Arg AGG (CCU) differ only at position 35. That the same mutations arise in selections for improving the function of two very similar anticodon loops is not surprising.

Importantly, the orthogonality of the *M. jannaschii* tRNA_CCU_-C3/TyrRS-C3 and *M. jannaschii* tRNA_CCU_-G4/TyrRS-G4 variants was maintained. Incorporation of tyrosine in response to an AGG codon using orthogonal translation machinery vectors from which the tRNA was removed was below the limit of detection of the fluorescence-based screen (0.2%, 2 of every 1000 incorporation events in response to the AGG codon in the reporter). The anticodon is often an important identity element for aaRSs, and the *M. jannaschii* TyrRS is known to utilize the anticodon during the process of aminoacylation. The most likely way in which orthogonality of the pair might be broken after mutation of the aaRS anticodon binding domain to better recognize the tRNA_CCU_ would be aminoacylation of Tyr onto the *E. coli* tRNA_CCU_. The absence of detectable fluorescence in vectors expressing either TyrRS-C3 or TyrRS-G4 without their cognate tRNA suggests that is not a frequent occurrence. These two aaRS variants were selected for the verification of orthogonality because they represent the 2 broad sequence spaces identified in the characterized clones.

As with our previous reports of sense codon reassignment using the fluorescence-based screen, the fluorescence measurements from the in cell assay were compared to fluorescence measurements from isolated protein. GFP protein production across the different AGG reassigning systems and non-reassigning controls is remarkably similar (Supplementary Figure S2). In this report, clarified lysate fluorescence measurements were normalized to protein amounts determined from image quantification of SDS-PAGE gels. Reassignment efficiency of AGG to tyrosine was identical within error using both types of measurement. Analysis of clarified lysates showed that *M. jannaschii* tRNA_CCU_/TyrRS was 60.7 ± 2.4% efficient and that *M. jannaschii* tRNA_CCU_-C3/TyrRS-C3 was 96.0 ± 2.0% efficient. The protein reassignment efficiencies from clarified lysates comprise at least 4 biological replicates of each system.

Reassignment efficiencies are calculated using the optical density-corrected fluorescence over a 4 h period of cell growth in order to eliminate biases from differences in the growth profiles of different codon reassigning systems. Bacterial cells are largely tolerant of induced missense mutations. We have previously discussed the average impact of high level codon reassignment on cellular fitness (Schmitt et al., 2018; Schwark et al., 2020). Using these orthogonal translation machinery and reporter vectors, the average fitness reduction for 20 different sense codon reassigning systems is about 2.5x the fitness reduction imposed by using 2 antibiotics to maintain 2 different protein expressing plasmids in the cells. The cell strain used for sense codon reassignment evaluation in the majority of our prior work was SB3930. Our recent reports have utilized the more commonly-available laboratory strain DH10B (Schwark et al., 2021). Codon reassignments have similar impacts on cell health in both strains.

Reassignment of Arg AGG using *M. jannaschii* tRNA_CCU_/TyrRS results in reduced carrying capacity and instantaneous doubling times in DH10B relative to an “empty” translation machinery vector from which both the tRNA and aaRS gene cassettes have been deleted. *E. coli* DH10B expressing *M. jannaschii* no-tRNA/no-aaRS and the GFP reporter vector with a Tyr codon in the fluorophore have an instantaneous doubling time of 40.0 ± 3.0 min, while those reassigning the AGG codon to tyrosine have an instantaneous doubling time of 45.8 ± 2.3 min. The relative system fitness of the AGG reassigning system is ∼87% that of the “empty” translation machinery control. Interestingly, expression of *M. jannaschii* tRNA_CCU_-C3/TyrRS-C3 restores cell fitness back to that of the “empty” translational machinery vector control. *E. coli* DH10B reassigning the AGG codon with *M. jannaschii* tRNA_CCU_-C3/TyrRS-C3 double every 36.7 ± 2.2 min in the exponential phase, for a relative system fitness of ∼109%. The tolerance toward high level reassignment of Arg AGG codons is expected to partly be the result of the low usage of the AGG codon throughout the *E. coli* genome. A representative optical density *vs.* time cell growth graph is provided as Supplementary Figure S3.

### Mutations That Improve Incorporation of Tyr in Response to AGG Also Improve Incorporation of a ncAA

**FIGURE 3:**
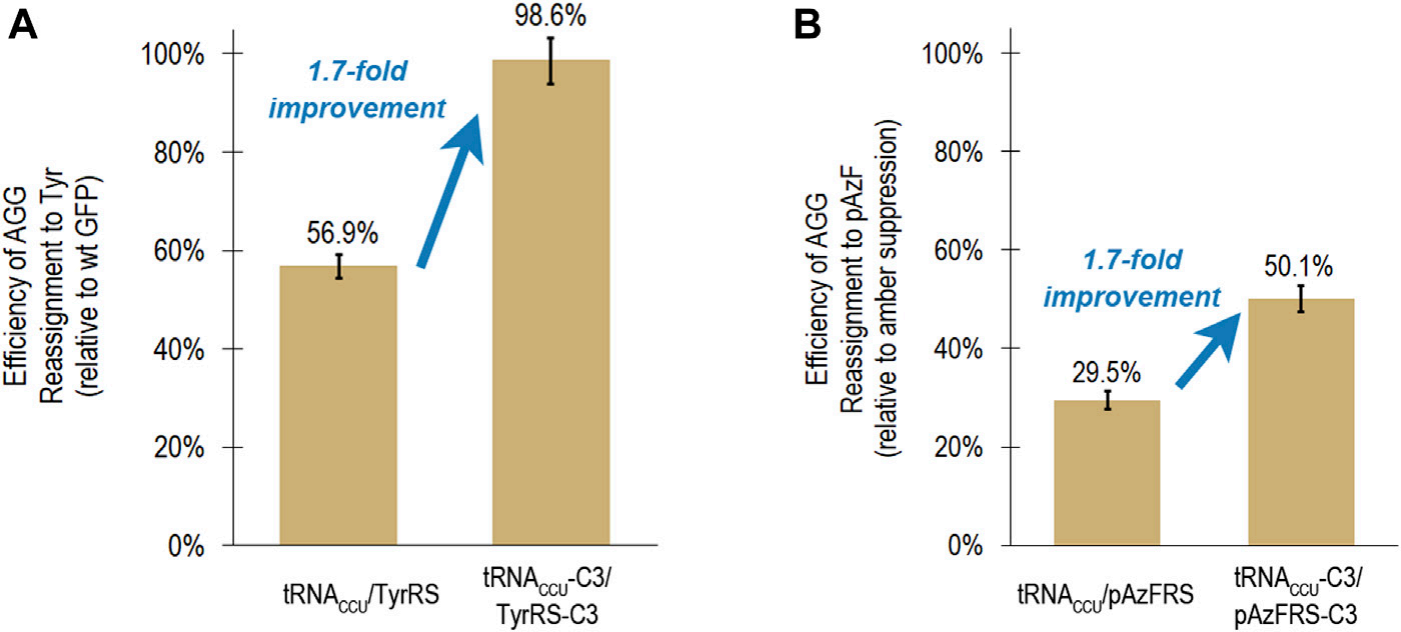
Transferability of mutations that led to increased reassignment of the AGG codon to tyrosine to an aaRS capable of incorporating a ncAA (*para*-azidophenylalanine). In both cases, the same suite of mutations to the tRNA anticodon loop and aaRS anticodon binding domain resulted in a 1.7-fold improvement in reassignment efficiency. **(A)** Efficiency of AGG sense codon reassignment to tyrosine by two orthogonal pairs: *M. jannaschii* tRNA_CCU_/TyrRS and tRNA_CCU_-C3/TyrRS-C3. Reported efficiencies are the mean and standard deviation for 24 biological replicates of each system evaluated in several independent iterations of the fluorescence-based screen. Reassignment efficiencies are corroborated by fluorescence analysis of the GFP protein mixtures isolated from these cells. **(B)** Efficiency of AGG sense codon reassignment to a ncAA, pAzF, by two orthogonal pairs: *M. jannaschii* tRNA_CCU_/pAzFRS and tRNA_CCU_-C3/pAzFRS-C3. Reported efficiencies are the mean and standard deviation of at least 4 biological replicates of each system quantified using the fluorescence per protein of GFP mixtures isolated from these cells. The “100% pAzF incorporation” control in each experiment is based on the fluorescence per protein analysis of full length GFP proteins produced after suppression of an amber stop codon in the fluorophore position 66.

As part of a general process for directed evolution of sense codon reassigning systems, the fluorescence-based screen with the tyrosine-incorporating aaRSs would be utilized as a stand in for aaRS variants that activate ncAAs. A group of clones with different sequences (C3, H1, and G4) exhibited very high, near quantitative reassignment of AGG to Tyr using the fluorescence-based screen. The reassignment efficiencies for each of these systems were all within error of each other, based on evaluation of 12 or 24 biological replicates of each system. In order to evaluate the transferability of improvements in tRNA/aaRS interactions between aaRS variants that activate different amino acids, the tRNA anticodon loop and aaRS anticodon binding domain mutations present in clone C3 were transferred to an aaRS variant that activates *para*-azidophenylalanine (pAzFRS) and evaluated both using both the fluorescence-based screen and mass spectrometry (Chin et al., 2002; Kirshenbaum et al., 2002). The C3 mutations improved AGG reassignment to Tyr from 56.9 ± 2.4% to 98.6 ± 4.7% (Figure 3A).

The *M. jannaschii* pAzF incorporating variant has been employed to direct incorporation of pAzF in response to both amber stop and arginine AGG codons (Chin et al., 2002; Lee et al., 2015). pAzF has been widely used for protein crosslinking *via* photolysis and bioorthogonal derivatization *via* copper-catalyzed Huisgen cyclization with alkynes. pAzF is one of several ncAAs that produce fluorescent proteins when introduced at the fluorophore tyrosine position in GFP (Wang et al., 2003a; Kajihara et al., 2005). Introduction of pAzF into GFP results in a protein with less intense fluorescence and blue shifted maximum relative to sfGFP. The apparent brightness (quantum yield*extinction coefficient) of sfGFP with pAzF in the fluorophore is approximately 10% of wild type sfGFP (Wang et al., 2012; Reddington et al., 2013). The actual fluorescent species in GFP with pAzF substitution is the reduced form of pAzF, *para*-aminophenylalanine. Reports differ on the extent to which spontaneous reduction of the azide to an amine occurs in GFP (Wang et al., 2003a; Morris et al., 2013; Reddington et al., 2013). In biological contexts, azides are often subject to rapid reduction by common dithiols (Bayley et al., 1978; Staros et al., 1978).

The fluorescence of cells expressing both the AGG reassignment reporter and either the *M. jannaschii* tRNA_CCU_/pAzFRS or *M. jannaschii* tRNA_CCU_-C3/pAzFRS-C3 was monitored in both the presence and absence of pAzF. Meaningful *in vivo* fluorescence was not observed for these cultures, possibly due to the lower inherent brightness of *para*-aminophenylalanine-containing GFP, the absence of spontaneous reduction of the azide, or obfuscation of protein fluorescence by fluorescent components in the rich medium. A slight increase in fluorescence was observed after irradiation of the cell cultures with UV light; however, controlled irradiation of several cell cultures was challenging. Reduction of the azide side chain following protein isolation proved to be more controllable.

In order to use fluorescence to quantify ncAA incorporation efficiency, the differences in the photophysical properties of GFP with pAmF (reduced pAzF) in the fluorophore need to be included in the measurements. The 100% fluorescence control for reassignment to pAzF was the per protein fluorescence of pAzF incorporated in response to an amber stop codon in the fluorophore of GFP. Although the efficiencies of introduction of ncAAs in response to the amber stop codon using *M. jannaschii* tRNA/aaRS pair variants are typically in the 10–30% range relative to sense codons (Young et al., 2010), no protein is produced in the absence of ncAA. The protein isolated and subsequently quantified contains only the ncAA. The amount of protein was determined from SDS-PAGE gels using GFP standards to calibrate the visualization. The observed fluorescence was normalized to the protein amounts to derive a value for fluorescence per protein.

Based on fluorescence analysis of isolated proteins, *M. jannaschii* tRNA_CCU_/pAzFRS incorporated pAzF in response to the AGG codon in the fluorophore of GFP with 29.5 ± 0.1% efficiency in rich media. The *M. jannaschii* tRNA_CCU_-C3/pAzFRS-C3 orthogonal pair incorporated pAzF in response to the AGG codon with 50.1 ± 1.1% efficiency, a 1.7-fold improvement over the original *M. jannaschii* tRNA_CCU_/pAzFRS (Figure 3B). This same relative improvement was observed between *M. jannaschii* tRNA_CCU_/TyrRS (56.9 ± 2.4%) and *M. jannaschii* tRNA_CCU_-C3/TyrRS-C3 (98.6 ± 4.7%) (Figure 3A). The improved reassignment efficiency observed for the C3 variant is expected to be the result of increased effective concentration of aminoacylated tRNA due to improved recognition between the anticodon of the tRNA and the aaRS. In this instance, improvements made to the *M. jannaschii* tRNA/aaRS pair for incorporation one amino acid, Tyr, also increased the efficiency of incorporating another amino acid, pAzF, in response to AGG codons.

Electrospray ionization mass spectrometry (ESI-MS) was used to evaluate the levels of incorporation of arginine, tyrosine, and pAzF in response to a single AGG codon in the Z domain of protein A (Braisted and Wells, 1996). The Z domain is a small, soluble, 8.3 kDa, three-helix protein that has been employed as a reporter for stop codon suppression (Xie et al., 2007). The phenylalanine codon at position 5 in the native Z domain, a known permissive site, was mutated to AGG. The AGG codon containing Z domain variant was expressed in DH10B cells also expressing the *M. jannaschii* machinery to reassign AGG codons to tyrosine or pAzF (tRNA_CCU_/TyrRS, tRNA_CCU_-C3/TyrRS-C3, tRNA_CCU_/pAzFRS, or tRNA_CCU_-C3/pAzFRS-C3). Z domain proteins were isolated and analyzed using ESI-MS of the intact protein followed by deconvolution of the mass spectra using the Maximum Entropy algorithm (MassHunter Software, Agilent Technologies).

The calculated mass of the Z domain protein with arginine at the single AGG codon is 8308 Da, and the +14 Da peak observed for all parent peaks is likely due to a methylation of the Z domain (Stock et al., 1987; Apostol et al., 1995). The Z domain protein has been reported to be variously modified (Wang et al., 2003b; Zhang et al., 2003). Incorporation of tyrosine rather than arginine in response to the AGG codon results in a +7 Da shift. Incorporation of pAzF rather than arginine in response to the AGG codon results in a either a +6 or +32 Da shift (for the reduced amine or azide form of the amino acid, respectively).

**FIGURE 4:**
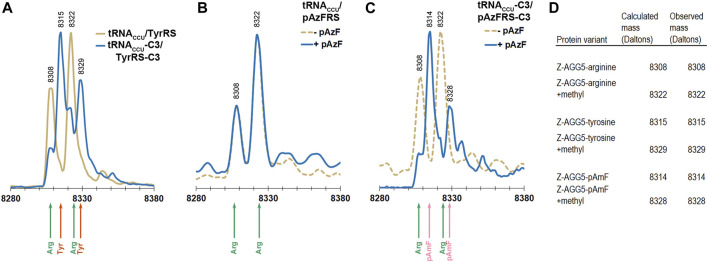
ESI-MS of purified Z domain proteins for identification of amino acids incorporated in response to an AGG codon at position 5 in the gene. The expected mass for incorporation of Arg in response to AGG is 8308 Da. The expected mass for incorporation of Tyr in response to AGG is 8315 Da. The expected mass for incorporation of reduced pAzF (pAmF) in response to AGG is 8314 Da. In all 3 instances, an additional mass at +14 Da relative to the parent mass is observed and likely corresponds to methylation of the Z domain. **(A)** Mass spectra for proteins produced using the tyrosine-incorporating *M. jannaschii* tRNA_CCU_/TyrRS pair (gold line) and *M. jannaschii* tRNA_CCU_-C3/TyrRS-C3 (blue line) variants. In the case of the original *M. jannaschii* tRNA_CCU_/TyrRS pair, only peaks for arginine incorporation are apparent (8308, 8322 Da, green arrows). Peaks at 8315 and 8329 Da (orange arrows) corresponding to tyrosine incorporation in response to AGG are readily apparent in the Z domain expressed in the presence of the *M. jannaschii* tRNA_CCU_-C3/TyrRS-C3 variant. **(B)** Mass spectra for proteins produced using the *para*-azidophenylalanine-incorporating *M. jannaschii* tRNA_CCU_/pAzFRS machinery in the presence (blue line) and absence (gold dotted line) of ncAA. In both cases, incorporation of Arg in response to AGG is the only set of masses detected (green arrows). **(C)** Mass spectra for proteins produced using the *para*-azidophenylalanine-incorporating *M. jannaschii* tRNA_CCU_-C3/pAzFRS-C3 machinery in the presence (blue line) and absence (gold dotted line) of ncAA. Only in the presence of *para*-azidophenylalanine are peaks corresponding to incorporation of ncAA observed at 8314 and 8328 Da (pink arrows). In the absence of ncAA, only arginine incorporation is detected (green arrows). **(D)** Table of calculated and observed masses (Daltons) for each protein.

Reassignment of the AGG codon to either tyrosine or pAzF was not detectable using the original forms of the *M. jannaschii* translation machinery where only the anticodon of the orthogonal tRNA was modified (tRNA_CCU_/TyrRS or tRNA_CCU_/pAzFRS). Masses corresponding to arginine incorporation were observed (Figures 4A,B). AGG reassignment is apparent in the deconvoluted mass spectra of cells expressing either tRNA_CCU_-C3/TyrRS-C3 or tRNA_CCU_-C3/pAzFRS-C3 (Figures 4A,C). In the case of protein isolated from cells expressing tRNA_CCU_-C3/TyrRS-C3, the peaks corresponding to arginine incorporation (8308 and 8322 Da) shrink, and peaks corresponding to tyrosine incorporation (8315 and 8329 Da) become evident. A similar trend is observed for proteins isolated from cells expressing tRNA_CCU_-C3/pAzFRS-C3 in the presence of pAzF.

The apparent difference in reassignment efficiencies in the Z domain protein as measured by ESI-MS and the GFP fluorescence-based screen is consistent with large observed variations in the efficiency of incorporation measured at different positions within the same protein and between various reporter proteins (Young et al., 2010). The efficiency of stop codon reassignment is thought to be sequence dependent based largely on differential interactions of release factors and suppressor tRNAs with regions outside the codon, codon context effects, additionally modulated by mRNA structure (Bossi and Roth 1980; Chevance et al., 2014; Gamble et al., 2016; Schwark et al., 2018). The extent to which codon context affects the rate and efficiency of sense codon reading and reassignment has not been widely investigated. While the absolute reassignment efficiencies are decidedly different in the two reporter protein systems, the trends in both suggest that tRNA/aaRS variants selected from a library that improve reassignment of AGG codons to tyrosine are transferable and increase the reassignment of AGG codons to pAzF.

### Increased Efficiencies Observed After Directed Evolution are Not Additive With Genomic Modification

The increase in sense codon reassignment efficiency that results from modifying interactions between the orthogonal tRNA/aaRS pair is expected to be independent from and potentially combinable with improvements that result from removing competition from endogenous tRNAs through genomic engineering. As near-quantitative reassignment of the AGG codon to tyrosine was observed using the *M. jannaschii* tRNA_CCU_-C3/TyrRS-C3, genomic engineering was not expected to markedly improve this system. Our evaluation of the effect of host strain genomic engineering on sense codon reassignment began instead with *M. jannaschii* tRNA_CCU_/TyrRS, which reassigns AGG to tyrosine with 56.9 ± 2.4% efficiency in *E. coli* DH10B.

One of the simplest genome modifications that may affect decoding the AGG codon is removal of the gene which encodes the *E. coli* rare arginine tRNA_CCU_, *argW*. The *argW* knockout of *E. coli* DH10B was prepared *via* the lambda red recombinase method (DS-dArgW) (Datsenko and Wanner, 2000). The strain mimics the previously-reported BS01 strain (DH10B Δ*arg*W) evaluated for reassignment of the AGG codon (Lee et al., 2015). An *argW/argA* double knockout named DS-dArgWArgA was also evaluated. The secondary *argA* knockout renders the cells auxotrophic for arginine. Sanger sequencing of the products of PCR amplifications of relevant segments of the chromosome and tRNA amplification tests indicated that the genomic knockouts were successful.

Eliminating competition for the AGG codon by removing the endogenous tRNA with a CCU anticodon resulted in a 1.6-fold *decrease* in measured reassignment efficiency. The *M. jannaschii* tRNA_CCU_/TyrRS reassigned AGG to tyrosine with 37.3 ± 0.3% efficiency in *E. coli* DS-dArgWArgA (12 biological replicates), compared to 56.9 ± 2.4% in *E. coli* DH10B. The observation that knocking out the competing tRNA was detrimental, as opposed to neutral or even beneficial, to sense codon reassignment efficiency was surprising. Cells from the original report (BS02, DH10B Δ*arg*W Δ*arg*A) were obtained for comparison to the DS-dArgWArgA strain and yielded similar results with our orthogonal machinery (Lee et al., 2016).

The finding that expression of the minor *E. coli* tRNA_CCU_ is not a major factor in allowing high level sense codon reassignment at the AGG codon is consistent with several other reports. Zeng et al. attempted to reduce the effective concentration of *E. coli* tRNA_CCU_ by introducing an antisense RNA and found that it had a no effect on sense codon reassignment efficiency (Zeng et al., 2014). Mukai et al. reported cells that *E. coli* cells were viable after knocking out *argW*, but were unable to survive after knocking out the genes for both the *E. coli* tRNA_CCU_ and *E. coli* tRNA_UCU_, which is able to read both the Arg AGA and AGG codons (Mukai et al., 2015). Supplemental information in Lee et al. suggests that very high levels of AGG codon reassignment are present in *argW* ^+^ parent cells consistent with the AGG reassignment efficiencies measured by the GFP screen (Lee et al., 2016). Direct comparisons between these systems are difficult as the vectors employed to produce both the orthogonal machinery and reporter proteins differ significantly across the reports. Preliminary evaluation of combinations of the variously evolved versions of orthogonal machinery expressed from different plasmid vectors (e.g. origin of replication, antibiotic resistance marker, and the promoters driving expression of the aaRS and tRNA) in the wildtype and knockout cell lines utilizing both fluorescence and mass reporters produced confounding results. The general conclusion that can be drawn from these experiments is that the systemic context in which a set of improvements in orthogonal system performance is selected is important. Combinations of improvements selected under different conditions do not necessarily add in predictable ways.

## Conclusion

We have described a general directed evolution pipeline for tailoring the tRNA/aaRS interactions of already-evolved ncAA-activating aaRSs for improved sense codon reassignment. Our pipeline exploits an easily screenable, tyrosine-activating aaRS as a stand in for ncAA-activating orthogonal aaRSs without readily screenable side chain chemistries. In the case of the ∼100 ncAA activating *M. jannaschii* orthogonal aaRS variants, the stand in aaRS is the natural, parent, tyrosine-activating *M. jannaschii* aaRS. This workflow should be applicable to ncAA-activating variants of the *M. barkeri* pyrrolysine aaRS as well, as we recently reported the evolution of a highly efficient tyrosine-incorporating variant of that pair (Schwark et al., 2021). We demonstrate the utility of the pipeline in improving the efficiency of *para*-azidophenylalanine incorporation in response to the arginine AGG codon. We selected variants that could more efficiently translate the AGG codon placed at the fluorophore tyrosine position in sfGFP from a library of *M. jannaschii* Tyr tRNA and aaRSs with focused mutations in the tRNA anticodon loop and aaRS tRNA anticodon binding domain. Translation of the codon at position 66 in a GFP reporter as either tyrosine or the amino acid typically specified by the target codon results in fluorescence signals proportional to the extent of successful reassignment. Screening large combinatorial libraries for improved variants is accomplished quickly *via* FACS. We showed that mutations that led to improved AGG codon reassignment identified using a tyrosine-activating aaRS also improved the efficiency of pAzF incorporation in response to the arginine AGG codon.

We expect that this directed evolution workflow will be readily applicable to improving incorporation of ncAAs in response to other targeted sense codons exploiting orthogonal tRNA/aaRS pairs already-evolved to recognize ncAAs and target suppression of an amber stop codon. Although decreasing competition from the endogenous translation machinery *via* genomic alterations proved effective for improving reassignment of the Arg AGG codon in other reports, the genomic alteration strategy is not necessarily readily transferrable to other attractive sense codon targets. Given the idiosyncrasies of *in vivo* protein translation and of the orthogonal pairs transplanted into it, improvements for one amino acid may not result in the same magnitude of improvement for another amino acid each time. However, we expect this approach to serve as a reasonable starting point for rapid identification of positional mutants with the likelihood of improving tRNA/aaRS recognition across aaRSs evolved for many amino acids.

## Data Availability

The raw data supporting the conclusion of this article will be made available by the authors, without undue reservation.
