# Supplementary material for: Directed Evolution Pipeline for the Improvement of Orthogonal Translation Machinery for Genetic Code Expansion at Sense Codons
Source: Front Chem. 2022 Feb 17;10:815788. doi: 10.3389/fchem.2022.815788 (PMC8891652; doi:10.3389/fchem.2022.815788)
Supplement: Supplementary file 1 [file DataSheet1.PDF]

Supplementary Material

TABLE OF CONTENTS

S.1: Cell strains	2
S.2: General reagents and materials	2
S.3: GFP reporter vectors for codon reassignment	2
S.4: *M. jannaschii* tRNA/aaRS pair expression vector backbone details and sequences	3
S.4.1 Sequence of the *M. jannaschii* TyrRS	3
S.4.2 Sequence of the *M. jannaschii* TyrRS-C3	3
S.4.3 Sequence of the *M. jannaschii* pAzFRS	3
S.4.4 Sequence of the *M. jannaschii* pAzFRS-C3	3
S.5: Preparation of ss dU DNA for site-directed mutagenesis	4
S.6: Preparation of an aaRS library via site-directed Kunkel mutagenesis	4
Table S1: Mutagenic oligonucleotides used for construction of the combined aaRS/tRNA anticodon loop library	5
Figure S1: Detailed directed evolution experimental workflow	6
S.7: Fluorescence-activated cell sorting for identification of tyrosine-incorporating aaRS variants	7
S.8: Workflow for verification of aaRS activity	7
S.9: GFP fluorescence-based codon reassignment assay protocol	8
S.10: Calculation of codon reassignment efficiencies	8
S.11: Protocol for protein isolation for verification of reassignment efficiency	9
Figure S2: Clarified lysate protein gel example	10
Figure S3: Representative optical density vs time plot for AGG reassigning systems	10
S.12: Expression and purification of Z domain proteins for mass spectrometry	11
S.12.1 Sequence of pSPEL253 protein expression vectors	11

S.1. Cell strains

DH10B (Invitrogen) F⁻ *mcr*A Δ(*mrr-hsd*RMS-*mcr*BC) φ80*lac*ZΔM15 Δ*lac*X74 *rec*A1 *end*A1 *ara*D139 Δ(*ara-leu*)7697 *gal*U *gal*K λ⁻ *rps*L(Str^R) *nup*G

CJ236 (New England Biolabs): FΔ*(HindIII)::cat* (Tra$^+$ Pil$^+$ Cam^R)/ *ung-1 relA1 dut-1 thi-1 spoT1 mcrA*

DS-dArgW: DH10B Δ*arg*W

DS-dArgWdArgA: DH10B Δ*arg*W Δ*arg*A

Both DS strains were prepared in house according to the method of Datsenko and Wanner Datsenko, K. A. and B. L. Wanner (2000). One-step inactivation of chromosomal genes in Escherichia coli K-12 using PCR products. *Proc. Natl. Acad. Sci. U. S. A.* 97(12): 6640-6645. DOI: 10.1073/pnas.120163297)

BS01: DH10B Δ*arg*W (Lee, B. S., S. Shin, J. Y. Jeon, K.-S. Jang, B. Y. Lee, S. Choi and T. H. Yoo (2015). Incorporation of Unnatural Amino Acids in Response to the AGG Codon. *ACS Chem. Biol.* 10(7): 1648-1653. DOI: 10.1021/acschembio.5b00230)

BS02: DH10B Δ*arg*W Δ*arg*A (Lee, B. S., S. Shin, J. Y. Jeon, K.-S. Jang, B. Y. Lee, S. Choi and T. H. Yoo (2015). Incorporation of Unnatural Amino Acids in Response to the AGG Codon. *ACS Chem. Biol.* 10(7): 1648-1653. DOI: 10.1021/acschembio.5b00230)

S.2 General materials and reagents

All restriction enzymes, DNA polymerases, and T4 kinase were purchased from New England Biolabs and used according to the manufacturer's instructions. ATP was purchased from Fisher (BP413-25) and dNTPs were purchased from New England Biolabs (N0447S). DNA isolation was performed using a Thermo Scientific GeneJET plasmid miniprep kit (K0503) according to the manufacturer's protocols. Intermediate cloning steps and PCR products were purified using a Thermo Scientific GeneJET PCR spin kit (K0701).

LB liquid media (per liter: 10 g tryptone, 5 g yeast extract, 5 g NaCl) and LB agar plates with 15 g/L agar (TEKNova, A7777) were used unless otherwise noted. Isopropyl-beta-D-thiogalactoside (IPTG) was purchased from Gold Bio (I2481C5). Spectinomycin (Enzo Life Science, BML-A281) was used at 50 μg/mL to maintain the vectors harboring the tRNA and aaRS genes. Carbenicillin (PlantMedia, 40310000-2) was used at 50 μg/mL to maintain the vectors harboring the GFP reporter gene. All bacterial cultures were grown at 37 °C unless otherwise noted. All liquid cultures were shaken at 225 rpm unless otherwise noted.

Electrocompetent stocks of all strains were prepared in-house according to the method of Sambrook and Russell (J. Sambrook and D. W. Russell *Molecular cloning: a laboratory manual*. **2001**, Cold Spring Harbor Laboratory press). Typical transformation efficiencies for electrocompetent cells produced in this way are 10^9 cfu/μg of supercoiled DNA. All transformations were recovered in SOC (20 g/L tryptone, 5 g/L yeast extract, 10 mM NaCl, 2.5 mM KCl, 10 mM $MgCl_2$, 20 mM glucose) for 1 hour at 37 °C with shaking prior to transfer to media containing appropriate antibiotics and/or inducers as noted.

All oligonucleotides were purchased from Integrated DNA Technologies (Coralville, Iowa, USA). DNA sequencing was performed by Genewiz (Plainfield, NJ, USA).

S.3 GFP reporter vectors for codon reassignment

Full sequence data for the suite of GFP reporter vectors used in this manuscript has been reported previously:

1) W. Biddle, M. A. Schmitt, and J. D. Fisk, Evaluating Sense Codon Reassignment with a Simple Fluorescence Screen, *Biochemistry*, **2015**, *54*, 7355-7364.
2) M. A. Schmitt, W. Biddle, and J. D. Fisk, Mapping the Plasticity of the *Escherichia coli* Genetic Code with Orthogonal Pair-Directed Sense Codon Reassignment. *Biochemistry*, **2018**, *57*, 2762-2774.
3) D. G. Schwark, M. A. Schmitt, W. Biddle, and J. D. Fisk, The Influence of Competing tRNA Abundance on Translation: Quantifying the Efficiency of Sense Codon Reassignment at Rarely Used Codons, *ChemBioChem*, **2020**, *21*, 2274-2286.

S.4 Vector backbone sequence details for the *M. jannaschii* orthogonal translation machinery

The vector backbone from which the *M. jannaschii* tRNA/aaRS variants are expressed for evaluation using the fluorescence-based screen is based on the vector used in our previous evaluations of sense codon reassignment by the *M. jannaschii* tRNA/aaRS orthogonal pair. The sequence of the entire vector was reported in W. Biddle, M. A. Schmitt, and J. D. Fisk, Evaluating sense codon reassignment with a simple fluorescence screen, *Biochemistry*, **2015**, *54*, 7355-7364.

Adjustments to the published vector sequence that are reported in this manuscript include the following:

S.4.1 Sequence of the parent, Tyr-incorporating aaRS variant:

Protein sequence: *M. jannaschii* Tyr aminoacyl tRNA synthetase

MDEFEMIKRNTSEIISEEELREVLKKDEKSAYIGFEPSGKIHLGHYLQIKKMIDLQNAGFDIIILLADLHAYLNQKGELDE
IRKIGDYNKKVFEAMGLKAKYVYGSEFQLDKDYTLNVYRLALKTTLKRARRSMELIAREDENPKVAEVIYPIMQVNDIHYL
GVDVAVGGMEQRKIHMLARELLPKKVVCIHNPVLTGLDGEGKMSSSKGNFIAVDDSPEEIRAKIKKAYCPAGVVEGNPIME
IAKYFLEYPLTIKRPEKFGGDLTVNSYEELESLFKNKELHPMDLKNAVAEELIKILEPIRKRL*

S.4.2 Sequence of the Tyr-incorporating aaRS variant C3:

Protein sequence: *M. jannaschii* Tyr aminoacyl tRNA synthetase

MDEFEMIKRNTSEIISEEELREVLKKDEKSAYIGFEPSGKIHLGHYLQIKKMIDLQNAGFDIIILLADLHAYLNQKGELDE
IRKIGDYNKKVFEAMGLKAKYVYGSEFQLDKDYTLNVYRLALKTTLKRARRSMELIAREDENPKVAEVIYPIMQVNDIHYL
GVDVAVGGMEQRKIHMLARELLPKKVVCIHNPVLTGLDGEGKMSSSKGNFIAVDDSPEEI**G**AKIK**N**A**YFQA**GVVEGNPIME
IAKYFLEYPLTIKRPEK**F**GGDLTVNSYEELESLFKNKEL**LRSG**LKNAVAEELIKILEPIRKRL*

*The amino acids in bold and highlighted in yellow were the 9 amino acids varied in the library.

**The amino acids in bold and highlighted in green were spontaneous mutations, at positions not varied in the library.

S.4.3 Sequence of the parent pAzF aaRS:

Protein sequence: *M. jannaschii para*-azidophenylalanine aminoacyl tRNA synthetase

MDEFEMIKRNTSEIISEEELREVLKKDEKSATIGFEPSGKIHLGHYLQIKKMIDLQNAGFDIIILLADLHAYLNQKGELDE
IRKIGDYNKKVFEAMGLKAKYVYGSNFQLDKDYTLNVYRLALKTTLKRARRSMELIAREDENPKVAEVIYPIMQVNPLHYQ
GVDVAVGGMEQRKIHMLARELLPKKVVCIHNPVLTGLDGEGKMSSSKGNFIAVDDSPEEIRAKIKKAYCPAGVVEGNPIME
IAKYFLEYPLTIKRPEKFGGDLTVNSYEELESLFKNKELHPMDLKNAVAEELIKILEPIRKRL*

S.4.4 Sequence of the pAzF-C3 aaRS:

Protein sequence: *M. jannaschii para*-azidophenylalanine aminoacyl tRNA synthetase with mutations from clone C3 identified using tyrosine-charging aaRS

MDEFEMIKRNTSEIISEEELREVLKKDEKSATIGFEPSGKIHLGHYLQIKKMIDLQNAGFDIIILLADLHAYLNQKGELDE
IRKIGDYNKKVFEAMGLKAKYVYGSNFQLDKDYTLNVYRLALKTTLKRARRSMELIAREDENPKVAEVIYPIMQVNPLHYQ
GVDVAVGGMEQRKIHMLARELLPKKVVCIHNPVLTGLDGEGKMSSSKGNFIAVDDSPEEI**G**AKIK**N**A**YFQA**GVVEGNPIME
IAKYFLEYPLTIKRPEK**F**GGDLTVNSYEELESLFKNKEL**LRSG**LKNAVAEELIKILEPIRKRL*

*The amino acids in bold and highlighted in yellow were the 9 amino acids varied in the library.

**The amino acids in bold and highlighted in green were spontaneous mutations, at positions not varied in the library, which were carried through when the mutations were mapped to the pAzF aaRS.

S.5. Preparation of ss dU DNA for site-directed Kunkel mutagenesis

The *M. jannaschii* tRNA/aaRS variant library and *M. jannaschii* tRNA anticodon variants were produced with Kunkel mutagenesis using a method adapted from Sidhu and Weiss (S. Sidhu and G. Weiss *Phage display: a practical approach* **2004** (Clackson, T. and Lowman, H.B., Ed.). Oxford University Press, USA). This mutagenic strategy relies upon preparation of single-stranded template DNA enriched in dU content. DNA is prepared in a cell strain lacking two of the enzymes responsible for editing deoxyuridine from newly synthesized DNA. Following annealing of mutagenic primers and extension of the template DNA in an in vitro reaction, the double-stranded DNA is transformed into cells that have the enzymes responsible for editing dU out of DNA. The non-mutated, template strand of DNA is degraded, and the mutated strand is replicated and transcribed.

Generic procedure for preparation of ss dU DNA: 4 mL cultures of CJ236 cells harboring the phagemid to be mutated were grown to an OD_{600} of 0.5 and infected with M13KO7 helper phage at a multiplicity of infection of 10:1. The infected culture was transferred into 125 mL of LB media with 5 μg/mL chloramphenicol, appropriate antibiotic to maintain the phagemid, and 0.25 μg/mL uridine. Cultures were grown overnight at 30 °C. Cells were pelleted at 17,000 x*g* at 4 °C for 20 minutes in a Sorvall RC 6+ with a Thermo FIBERLite F14-6x250y rotor. Phage particles were isolated by decanting the supernatant from the pelleted cells into $1/5^{th}$ volume of 20% 8,000 molecular weight polyethylene glycol and 2.5M NaCl in water. Solutions were incubated on ice for at least 2 hours. Phage particles were isolated by pelleting at 17,000 x*g* at 4 °C for 20 minutes. The supernatant was decanted, and the phage pellet was spun for an additional minute to collect the remaining supernatant, which was then removed. The phage pellet was resuspended in 1.5 mL of phosphate buffered saline, pH = 7.4. Insoluble material was pelleted out of the phage solution at 17,000 x*g* for 5 minutes. Single-stranded, uridine-enriched DNA (ss dU DNA) was isolated from phage using a Qiagen M13 spin kit.

S.6. Preparation of the site-directed tRNA/aaRS library

Four mutagenic primers (Table S1) were phosphorylated for 1.5 hours at 37 °C using T4 polynucleotide kinase. Phosphorylated primers were annealed to 5 μg of single-stranded template DNA at a 10:1 molar ratio by incubating at 90 °C for 2 minutes, 60 °C for 20 seconds, followed by a decreasing temperature ramp of 1°C every 20 seconds until the temperature reached 25 °C. The reaction was held at 25 °C for 5 minutes. The annealed mixture was extended using T7 DNA polymerase in the presence of T4 DNA ligase and 670 μM ATP and 330 μM dNTPs (each) at room temperature overnight.

The crude mutagenesis reaction was purified using a PCR spin kit column. The entirety of the eluted purified DNA (55 μL) was transformed into electrocompetent *E. coli* DH10B to degrade the ss dU DNA template. Transformed cells were allowed to recover in 6 mL of SOC without antibiotics for 1 hour at 37 °C. Following recovery, a small aliquot was removed for plating onto LB agar/spectinomycin to determine the size of the library. ~10^9 unique clones were generated. Based on PCR analysis of several unique clones, more than 10^8 clones are expected to be fully mutated by library primers (XhoI sites in the starting material were not present in the PCR product.)

The remainder of the SOC recovery media was diluted into LB with spectinomycin (80 mL total volume) and grown for approximately 2 doublings based on the starting OD_{600} (~3 hours). Plasmid DNA was purified from the culture using 4 miniprep columns. 6 μg of this DNA was digested with

XhoI (NEB) for 1.5 hours at 37 °C to remove nonmutated and partially mutated DNA. The digested DNA was purified using 1 PCR spin column using 50 µL of sterile water for elution.

Purified, digested DNA was transformed into electrocompetent *E. coli* DH10B already harboring the GFP reporter plasmid with an AGG codon at position 66. Transformations were recovered in a total of 50 mL SOC for 1 hour. SOC recovery media was diluted into LB media with appropriate antibiotics and 1 mM IPTG to induce expression of both the aaRS variants and the GFP reporter (600 mL total volume) and grown overnight. The following morning, the library was aliquoted into volumes containing 0.3 ODs and frozen at -80 °C in 35% glycerol for FACS analysis.

Table S1: Mutagenic oligonucleotides used for construction of the combined aaRS/tRNA anticodon loop library

Primer name	Primer sequence
aars_230-233	CCCTCAACCACGCCGRMCKKANAAKMCGCTTTCTTGATCTTCGCAC
aaRS-261	CAGGTCGCCGCCDDNTTTCTCCGGGCGC
aaRS-283-286	CCACGGCGTTTTTCAGWBBVVWANVSHRCAGTTCTTTGTTCTTAAACAAGCTTTC
agg-ac-loop	GGCAGAACGGCGGANTCCTNNTCCGCATGGCAGG

Figure S1: Detailed directed evolution workflow.

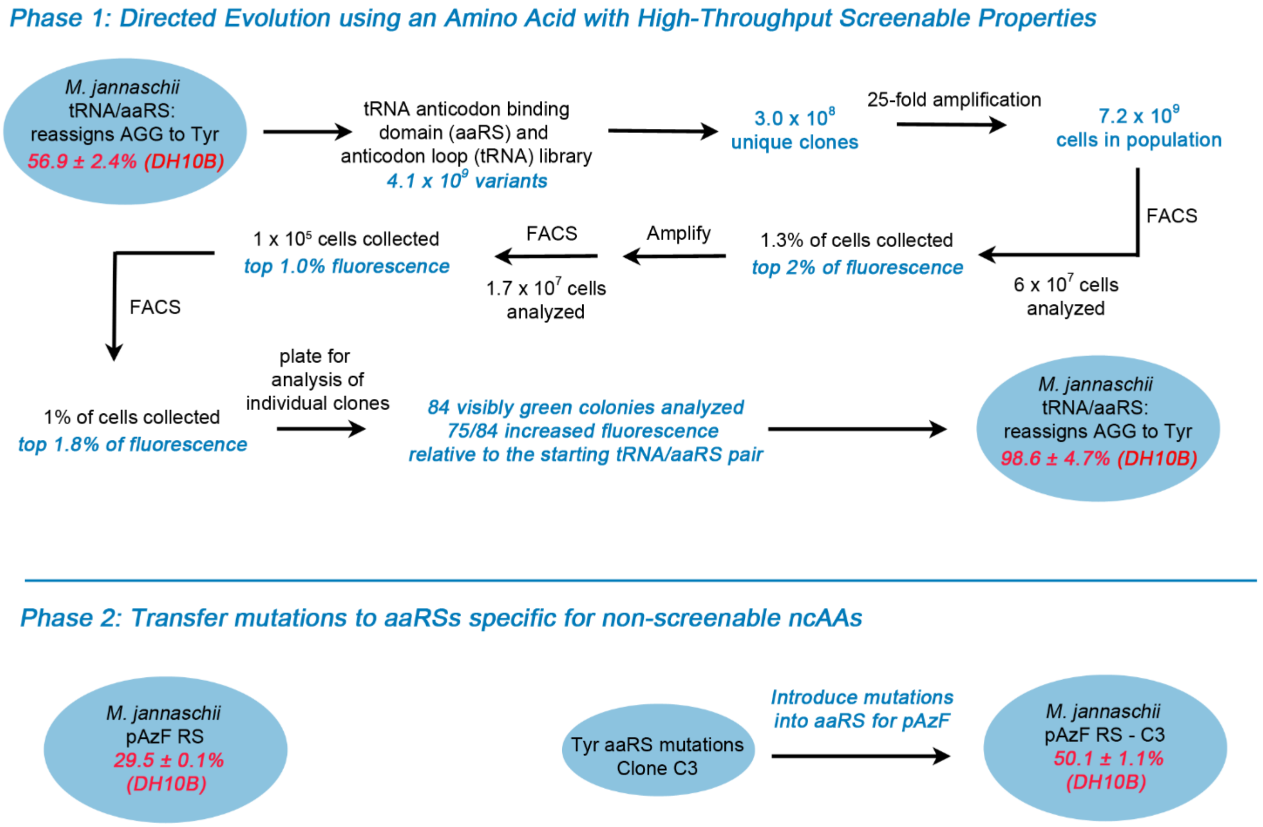

Supplementary Figure S1. Visual representation of the two-phase directed evolution workflow, including key quantifications (e.g. library size, FACS details, AGG reassignment efficiencies) for evolution of tyrosine-charging *M. jannaschii* aaRS to a variant that charges tyrosine to its cognate tRNA at an efficiency rivaling wild type tRNA/aaRS pairs and subsequent transfer of mutations to aaRSs specific for non (or less readily) screenable ncAAs.

S.7 Fluorescence-activated cell sorting (FACS) for identification of improved tyrosine-incorporating *M. jannaschii* tRNA/aaRS variants

Tubes of cells harboring the aaRS library and the AGG codon GFP reporter that had been stored at -80 °C in 35% glycerol were thawed on ice and centrifuged at 8,000 xg for 5 minutes at room temperature. The supernatant was removed via pipette, and 1 mL sterile 0.9% aqueous NaCl was added to each tube. Cells were resuspended via gentle pipetting and returned to ice.

FACS screening for identification of tyrosine-incorporating tRNA/aaRS variants with improved reassignment of Arg AGG was performed on a Dako-Cytomation MoFlo Legacy instrument using the 488 nm laser line with a 530/40 nm band-pass filter in front and the 70 μm flow cell tip. For all runs, cells were initially gated on forward and side scatter to remove clumps and non-cell particles. Cells were then gated on GFP fluorescence and sorting was performed on the purify 1,2 mode at approximately 21,000 events/second. Following sorting, collected cells were diluted directly into LB with appropriate antibiotics and IPTG to a final volume of 6 mL and grown overnight. The next day, aliquots of the saturated culture containing 0.3 ODs were stored at -80 °C in 35% glycerol for future use (e.g. additional FACS sorting).

Controls for FACS included wild type GFP (DH10B co-transformed with the GFP reporter with a Tyr codon at position 66 and the *M. jannaschii* tRNA/aaRS vector expressing the aaRS template for library synthesis) as well as a non-fluorescent control (DH10B co-transformed with a non-fluorescent GFP reporter (UAG at position 66) and the *M. jannaschii* tRNA/aaRS vector expressing the aaRS template for library synthesis). Prior to library evaluation, approximately 1.2 x 10^6 cells from each sample were analyzed to map the two control populations. Aliquots for flow cytometry were prepared in a fashion identical to that described for cells harboring library members.

Library sort #1: 6 x 10^7 cells were analyzed and 8 x 10^5 cells (~1.3% of total cells examined) with fluorescence representing the top 2% of clones were collected into LB. Cells were amplified by diluting collected fractions into 10x volume LB spec 50 carb 50 and allowed to grow overnight.

Library sort #2: 2.3 x 10^7 cells amplified from the first sort were analyzed and 1.0 x 10^4 cells (top 0.1% of fluorescence) were collected. Cells were amplified as described above.

Library sort #3: 3 x 10^6 cells amplified from the second sort were analyzed and 2 x 10^4 cells were collected. Cells were amplified as described above, and a portion were plated for analysis of individual clones.

S.8. Workflow for verification of aaRS variant activity

Analysis of individual clones using the *in vivo* fluorescence-based screen precludes the inclusion of biological replicates. Following characterization of the performance of an individual clone, several additional evaluations are undertaken to confirm system behavior.

First, vector DNA is isolated from the individual clone. The DNA includes both the GFP reporter and the orthogonal translation machinery vectors. Restriction enzymes with recognition sites unique to the GFP reporter vector are used to separate the two vectors. Following digestion, the reaction is transformed into electrocompetent *E. coli* DH10B and plated onto LB agar containing either the antibiotic to which resistance is conferred by the orthogonal translation machinery vector (spectinomycin) or both spectinomycin and carbenicillin (the antibiotic upon which cells harboring intact GFP reporter vector can grow) to confirm the successful digestion of the GFP reporter vector. A single colony is grown up in ~8 mL LB/Spectinomycin, and the DNA is isolated and sequenced.

Isolated DNA is co-transformed with the appropriate GFP reporter vector, and multiple colonies are evaluated in the fluorescence-based screen. In this case, the GFP reporter vector DNA has

not been subjected to selection. The isolation and retransformation procedure provides an opportunity to evaluate biological replicates of a given system and to ensure that observed activity was not the result of spontaneous mutations to either vector (e.g. promoter mutations that increase gene expression).

In order to confirm that an identified aaRS sequence enables reassignment of a given codon in a particular system with the reported efficiency, the aaRS gene was PCR amplified out of the isolated DNA and recloned into the backbone vector. Again, the vector DNA used in the cloning reaction had not been through rounds screening and amplification and is not expected to have undesired mutations. The cloned DNA is isolated and sequenced to confirm that the sequence has not changed. This DNA is co-transformed with a GFP reporter vector with the codon of interest specifying the fluorophore tyrosine and evaluated in the fluorescence-based screen. Only after demonstrating consistent codon reassignment efficiency in each of these tests is an aaRS considered active.

S.9. GFP fluorescence-based sense codon reassignment assays

Superfolder green fluorescent protein (GFP) reporter plasmids were co-transformed with vectors expressing the modified orthogonal translational components into *E. coli* DH10B. After overnight growth, colonies were picked into 200 µL LB media in a 96 well plate. Cells were grown to at least mid-log phase (usually 8-10 hours) with shaking at 37 °C. Cells were diluted 10-fold into LB media with antibiotics to maintain the plasmids and 1 mM IPTG for induction of both the aaRS and GFP. Assays were performed in a Fluorotrac 200 clear bottom 96 well plate (Greiner 655096) and monitored in a BioTek Synergy H1 or BioTek Synergy Neo 2S plate reader at 37 °C with continuous double orbital shaking. The optical density (OD600) and fluorescence of each well was measured every 15 minutes for at least 15 hours; optical density was measured at 600 nm, and fluorescence was measured with an excitation at 485 nm and detection at 515 nm with a 20 nm band pass.

S.10 Calculation of sense codon reassignment efficiency from optical density and fluorescence readings

For each biological replicate, the relative fluorescence (corrected fluorescence per OD) was calculated for each of the 16 data points gathered between 8 and 12 hours after induction of GFP and the aaRS with IPTG. The 16 relative fluorescence values were averaged to determine the RFU for each sample. That RFU is divided by the average RFU for all biological replicates of the 100% reassigning fluorescence control (wild type GFP) to determine the reassignment efficiency. Sense codon reassignment efficiency for each tRNA anticodon/GFP codon variant pair was calculated by averaging the reassignment efficiency for at least six biological replicates. An extremely detailed discussion and workflow for calculating codon reassignment efficiency was provided in the Supporting Information for M. A. Schmitt, W. Biddle, and J. D. Fisk, Mapping the Plasticity of the *Escherichia coli* Genetic Code with Orthogonal Pair-Directed Sense Codon Reassignment. *Biochemistry*, **2018**, *57(19)*, 2762-2774.

All reported codon reassignment efficiencies represent the mean and standard deviation of at least 12 biological replicates. Reported AGG reassignment efficiencies for *M. jannaschii* $tRNA_{CCU}$/TyrRS and $tRNA_{CCU}$-C3/TyrRS-C3 efficiencies are the mean and standard deviation of 24 biological replicates.

S.11 Protein isolation for verification of reassignment efficiency

Tyr-incorporating and pAzF-incorporating orthogonal translation machinery vectors were evaluated in combination with relevant GFP reporter vectors to evaluate protein production. For Tyr-incorporating variants, cells evaluated using the in cell, fluorescence-based screen were lysed and evaluated, to allow direct comparison of in cell reassignment efficiency calculations with those from the isolated GFP proteins. The clarified lysates from these cells contain a mixture of both Arg and Tyr containing GFP proteins.

Cells were grown as described for the fluorescence-based screen. Following completion of the in cell assay, the same number of cells from each system (volumes of cultures were normalized based on the optical density of each well from which protein was to be isolated) were pelleted in a microcentrifuge tube by spinning at maximum speed (17,000 xg) for 5 minutes at room temperature. Following removal of the spent media, cell pellets were frozen at -20C overnight. The following day cell pellets were lysed using B-PER (ThermoFisher Scientific) according to the manufacturer's instructions. Lysates were clarified by spinning at 17,000 xg for 20 minutes at 4C. Cleared lysates were transferred to a new microfuge tube away from cellular debris.

Cleared lysates were analyzed by SDS-PAGE and stained with Coomasie (BioRad) according to the manufacturer's instructions. Protein amounts were estimated using ImageJ (https://imagej.nih.gov/ij/) and the band intensities for four GFP standards (110ng, 225 ng, 550 ng, and 1100 ng loaded). The fluorescence of each sample was quantified using either the BioTek Synergy H1 or BioTek Synergy Neo 2S plate reader. The observed fluorescence was normalized to the protein amount to derive a value for fluorescence per protein.

An identical procedure was followed for the pAzF-incorporating machinery. Fluorescence per protein measurements were used to calculate the reassignment efficiency of AGG to pAzF. A sample of protein isolated from cells expressing the orthogonal machinery designed to charge pAzF onto a tRNA with a CUA anticodon for amber stop suppression served as the 100% reassignment control. In order to use fluorescence to quantify the ncAA incorporation efficiency, the differences in the photophysical properties of GFP with pAmF (reduced pAzF) in the fluorophore need to be included in the measurements. As such, a fluorescence per protein for the 100% fluorescence that has exclusively pAmF in the fluorophore is needed. Although protein yields from stop codon suppression are lower than endogenous translation of a sense codon, fluorescence measurements were normalized to the amount of protein based on SDS-PAGE image analysis.

Figure S2: Representative gel image of clarified lysates from cells with various orthogonal translation machinery components and GFP reporters

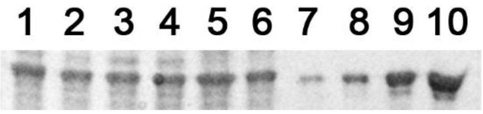

Representative Coomasie stained SDS-PAGE analysis of clarified cell lysates for DH10B cells harboring various orthogonal translation machinery components

Lane 1: $tRNA_{CCU}$/TyrRS and Arg AGG GFP reporter (AGG codon at position 66)

Lane 2: $tRNA_{CCU}$/TyrRS and wild type sf GFP reporter (Tyr codon at position 66)

Lane 3: $tRNA_{CCU}$-C3/TyrRS-C3 and Arg AGG GFP reporter (AGG codon at position 66)

Lane 4: $tRNA_{CCU}$-C3/TyrRS-C3 and wild type sf GFP reporter (Tyr codon at position 66)

Lane 5: $tRNA_{CUA}$/TyrRS and amber stop GFP reporter (TAG codon at position 66)

Lane 6: $tRNA_{CUA}$/TyrRS and wild type sf GFP reporter (Tyr codon at position 66)

Lanes 7-10: purified GFP mass controls: 110ng, 225 ng, 550 ng, and 1100 ng, respectively

Figure S3: Representative optical density vs time graph for AGG reassigning systems

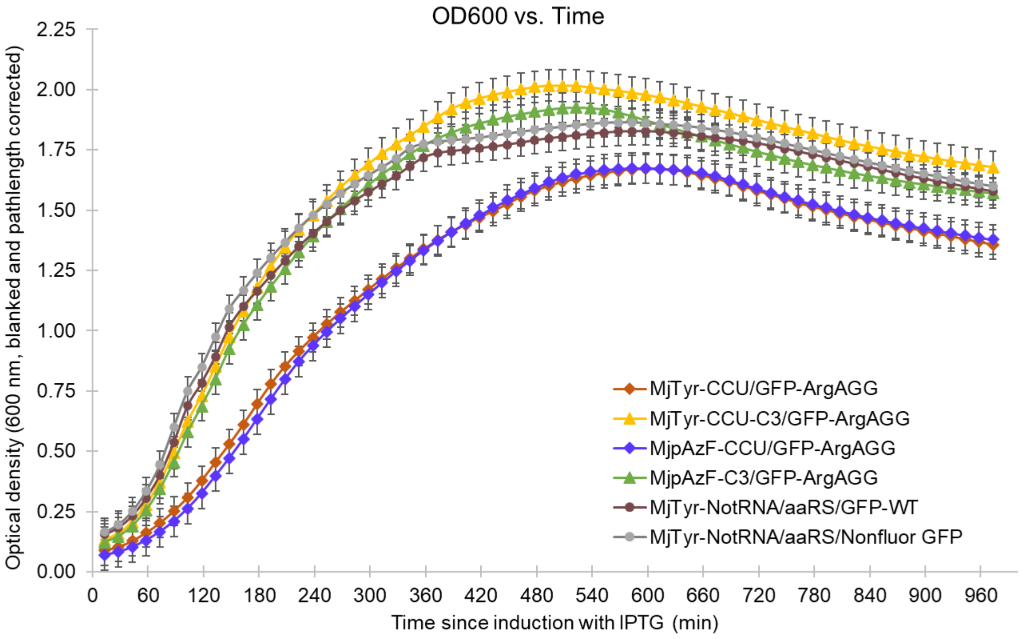

Figure S3. Optical density versus time profile for systems reassigning the AGG sense codon. Growth profiles are discussed relative to DH10B cells expressing a GFP reporter (either wildtype GFP or a non-fluorescent variant) and an “empty” translation machinery vector from which the orthogonal tRNA and aaRS have been removed. Reassignment of the AGG codon using the starting tRNA/aaRS sequences decreases both the carrying capacity and instantaneous doubling time of the cells. Reassignment of the AGG codon using the improved C3 tRNA/aaRS variant restores cellular fitness. Means and standard deviations are for 6 biological replicates of each system.

S.12 Expression and purification of Z domain proteins for mass spectrometry analysis

Z domain of protein A reporter plasmids pSPEL253-F5AGG were co-transformed into DH10B with vectors containing the four main orthogonal engineered *M. jannaschii* translational components: *M. jannaschii* $tRNA_{CCU}$ /TyrRS, *M. jannaschii* $tRNA_{CCU}$-C3/TyrRS-C3, *M. jannaschii* $tRNA_{CCU}$ /pAzFRS, or *M. jannaschii* $tRNA_{CCU}$-C3/pAzFRS-C3. Three isolated colonies of each system were grown overnight in ~8 mL volume (LB with appropriate antibiotics, and, in the case of the 2 pAzF aaRS vectors, *para*-azidophenylalanine). Overnight cultures were diluted to a starting OD_{600} of approximately 0.1 in 40 mL pre-warmed LB with antibiotics and IPTG to express the engineered aaRS and the reporter protein. Protein expression proceeded for 12 hours at 37 °C and 225 rpm. 30ml of 1 OD/ml of cells were pelleted at 17,000 x *g*, 4 °C for 30. The supernatant was discarded, and the cell pellets were frozen at -20 °C overnight. The following morning, cell pellets were thawed at room temperature, and 1.8 mL of B-Per lysis reagent (Thermo Scientific 78248, prepared according to manufacturer's instructions) was added to each cell pellet. After 30 minutes at room temperature, the lysed cell culture was clarified by centrifugation at 17,000 x *g*, 4 °C for 1 hour. Clarified lysate for each sample was transferred to a clean tube. 20 µL of each sample was saved for SDS-PAGE analysis.

The Z domain of protein A was then purified from the remaining amount of clarified lysate for each sample using Qiagen Ni-NTA spin columns (Qiagen 31014) according to the manufacturer's instructions. 20 µL from each step in the purification process was saved for SDS-PAGE analysis. Bound protein was eluted twice with 2 successive 250 µL volumes of NPI-500 buffer (500 µL total). Protein samples were buffer-exchanged into HPLC-grade water using a 3,000 molecular weight cutoff column according to manufacturer's instructions (Millipore UF500396). The final volume of each protein sample after buffer exchange was 300 µL.

ESI-MS was performed in the Chemistry Instrumentation Facility at Colorado State University. Spectra were deconvoluted using the Maximum Entropy algorithm and MassHunter Software from Agilent Technologies.

S.12.1 DNA sequence for pSPEL253-F5AGG protein expression vector

The pSPEL253 Z domain protein expression vector pSPEL253-F5AGG was a generous gift from the laboratory of Tae Hyeon Yoo. (Reference: B. S. Lee, S. Seunggun, J. Y. Jeon, K-S. Jang, B. Y. Lee, S. Choi, T. H. Yoo, Incorporation of Unnatural Amino Acids in Response to the AGG Codon, *ACS Chem Biol*, **2015**, *10*, 1648-1653)

The sequence of the Z domain protein with a single AGG codon targeted for reassignment is:

ATGAGAGGATCGCATCACCATCACCATCACGGATCCATGGCCGTAGACAACAAA**AGG**AACAAAGAAC
AACAAAACGCGTTCTATGAGATCTTACATTTACCTAACTTAAACGAAGAACAACGAAACGCCTTCAT
CCAAAGTTTAAAAGATGACCCAAGCCAAAGCGCTAACCTTTTAGCAGAAGCTAAAAAGCTAAATGAT
GCTCAGGCGCCGTGG

The sequence of the entire pSPEL253-F5AGG vector is:

CTCGAGAAATCATAAAAAATTTATTTGCTTTGTGAGCGGATAACAATTATAATAGATTCAATTGTGA
GCGGATAACAATTTCACACAGAATTCATTAAAGAGGAGAAATTAACTATGAGAGGATCGCATCACCA
TCACCATCACGGATCCATGGCCGTAGACAACAAAAGGAACAAAGAACAACAAAACGCGTTCTATGAG
ATCTTACATTTACCTAACTTAAACGAAGAACAACGAAACGCCTTCATCCAAAGTTTAAAAGATGACC
CAAGCCAAAGCGCTAACCTTTTAGCAGAAGCTAAAAAGCTAAATGATGCTCAGGCGCCGTGGTAAAA
GCTTAATTAGCTGAGCTTGGACTCCTGTTGATAGATCCAGTAATGACCTCAGAACTCCATCTGGATT
TGTTCAGAACGCTCGGTTGCCGCCGGGCGTTTTTTATTGGTGAGAATCCAAGCTAGCTTGGCGAGAT
TTTCAGGAGCTAAGGAAGCTAAAATGGAGAAAAAAATCACTGGATATACCACCGTTGATATATCCCA
ATGGCATCGTAAAGAACATTTTGAGGCATTTCAGTCAGTTGCTCAATGTACCTATAACCAGACCGTT

CAGCTGGATATTACGGCCTTTTTAAAGACCGTAAAGAAAAATAAGCACAAGTTTTATCCGGCCTTTA
TTCACATTCTTGCCCGCCTGATGAATGCTCATCCGGAATTTCGTATGGCAATGAAAGACGGTGAGCT
GGTGATATGGGATAGTGTTCACCCTTGTTACACCGTTTTCCATGAGCAAACTGAAACGTTTTCATCG
CTCTGGAGTGAATACCACGACGATTTCCGGCAGTTTCTACACATATATTCGCAAGATGTGGCGTGTT
ACGGTGAAAACCTGGCCTATTTCCCTAAAGGGTTTATTGAGAATATGTTTTTCGTCTCAGCCAATCC
CTGGGTGAGTTTCACCAGTTTTGATTTAAACGTGGCCAATATGGACAACTTCTTCGCCCCCGTTTTC
ACCATGGGCAAATATTATACGCAAGGCGACAAGGTGCTGATGCCGCTGGCGATTCAGGTTCATCATG
CCGTTTGTGATGGCTTCCATGTCGGCAGAATGCTTAATGAATTACAACAGTACTGCGATGAGTGGCA
GGGCGGGGCGTAATTTTTTTAAGGCAGTTATTGGTGCCCTTAAACGCCTGGGGTAATGACTCTCTAG
CTTGAGGCATCAAATAAAACGAAAGGCTCAGTCGAAAGACTGGGCCTTTCGTTTTATCTGTTGTTTG
TCGGTGAACGCTCTCCTGAGTAGGACAAATCCGCCCTCTAGATTACGTGCAGTCGATGATAAGCTGT
CAAACATGAGAATTGTGCCTAATGAGTGAGCTAACTTACATTAATTGCGTTGCGCTCACTGCCCGCT
TTCCAGTCGGGAAACCTGTCGTGCCAGCTGCATTAATGAATCGGCCAACGCGCGGGGAGAGGCGGTT
TGCGTATTGGGCGCCAGGGTGGTTTTTCTTTTCACCAGTGAGACGGGCAACAGCTGATTGCCCTTCA
CCGCCTGGCCCTGAGAGAGTTGCAGCAAGCGGTCCACGCTGGTTTGCCCCAGCAGGCGAAAATCCTG
TTTGATGGTGGTTAACGGCGGGATATAACATGAGCTGTCTTCGGTATCGTCGTATCCCACTACCGAG
ATATCCGCACCAACGCGCAGCCCGGACTCGGTAATGGCGCGCATTGCGCCCAGCGCCATCTGATCGT
TGGCAACCAGCATCGCAGTGGGAACGATGCCCTCATTCAGCATTTGCATGGTTTGTTGAAAACCGGA
CATGGCACTCCAGTCGCCTTCCCGTTCCGCTATCGGCTGAATTTGATTGCGAGTGAGATATTTATGC
CAGCCAGCCAGACGCAGACGCGCCGAGACAGAACTTAATGGGCCCGCTAACAGCGCGATTTGCTGGT
GACCCAATGCGACCAGATGCTCCACGCCCAGTCGCGTACCGTCTTCATGGGAGAAAATAATACTGTT
GATGGGTGTCTGGTCAGAGACATCAAGAAATAACGCCGGAACATTAGTGCAGGCAGCTTCCACAGCA
ATGGCATCCTGGTCATCCAGCGGATAGTTAATGATCAGCCCACTGACGCGTTGCGCGAGAAGATTGT
GCACCGCCGCTTTACAGGCTTCGACGCCGCTTCGTTCTACCATCGACACCACCACGCTGGCACCCAG
TTGATCGGCGCGAGATTTAATCGCCGCGACAATTTGCGACGGCGCGTGCAGGGCCAGACTGGAGGTG
GCAACGCCAATCAGCAACGACTGTTTGCCCGCCAGTTGTTGTGCCACGCGGTTGGGAATGTAATTCA
GCTCCGCCATCGCCGCTTCCACTTTTTCCCGCGTTTTCGCAGAAACGTGGCTGGCCTGGTTCACCAC
GCGGGAAACGGTCTGATAAGAGACACCGGCATACTCTGCGACATCGTATAACGTTACTGGTTTCACA
TTCACCACCCTGAATTGACTCTCTTCCGGGCGCTATCATGCCATACCGCGAAAGGTTTTGCACCATT
CGATGGTGTCGGAATTTCGGGCAGCGTTGGGTCCTGGCCACGGGTGCGCATGATCTAGAGCTGCCTC
GCGCGTTTCGGTGATGACGGTGAAAACCTCTGACACATGCAGCTCCCGGAGACGGTCACAGCTTGTC
TGTAAGCGGATGCCGGGAGCAGACAAGCCCGTCAGGGCGCGTCAGCGGGTGTTGGCGGGTGTCGGGG
CGCAGCCATGACCCAGTCACGTAGCGATAGCGGAGTGTATACTGGCTTAACTATGCGGCATCAGAGC
AGATTGTACTGAGAGTGCACCATATGCGGTGTGAAATACCGCACAGATGCGTAAGGAGAAAATACCG
CATCAGGCGCTCTTCCGCTTCCTCGCTCACTGACTCGCTGCGCTCGGTCGTTCGGCTGCGGCGAGCG
GTATCAGCTCACTCAAAGGCGGTAATACGGTTATCCACAGAATCAGGGGATAACGCAGGAAAGAACA
TGTGAGCAAAAGGCCAGCAAAAGGCCAGGAACCGTAAAAAGGCCGCGTTGCTGGCGTTTTTCCATAG
GCTCCGCCCCCCTGACGAGCATCACAAAAATCGACGCTCAAGTCAGAGGTGGCGAAACCCGACAGGA
CTATAAAGATACCAGGCGTTTCCCCCTGGAAGCTCCCTCGTGCGCTCTCCTGTTCCGACCCTGCCGC
TTACCGGATACCTGTCCGCCTTTCTCCCTTCGGGAAGCGTGGCGCTTTCTCATAGCTCACGCTGTAG
GTATCTCAGTTCGGTGTAGGTCGTTCGCTCCAAGCTGGGCTGTGTGCACGAACCCCCCGTTCAGCCC
GACCGCTGCGCCTTATCCGGTAACTATCGTCTTGAGTCCAACCCGGTAAGACACGACTTATCGCCAC
TGGCAGCAGCCACTGGTAACAGGATTAGCAGAGCGAGGTATGTAGGCGGTGCTACAGAGTTCTTGAA
GTGGTGGCCTAACTACGGCTACACTAGAAGGACAGTATTTGGTATCTGCGCTCTGCTGAAGCCAGTT
ACCTTCGGAAAAAGAGTTGGTAGCTCTTGATCCGGCAAACAAACCACCGCTGGTAGCGGTGGTTTTT
TTGTTTGCAAGCAGCAGATTACGCGCAGAAAAAAAGGATCTCAAGAAGATCCTTTGATCTTTTCTAC
GGGGTCTGACGCTCAGTGGAACGAAAACTCACGTTAAGGGATTTTGGTCATGAGATTATCAAAAAGG
ATCTTCACCTAGATCCTTTTAAATTAAAAATGAAGTTTTAAATCAATCTAAAGTATATATGAGTAAA

CTTGGTCTGACAGTTACCAATGCTTAATCAGTGAGGCACCTATCTCAGCGATCTGTCTATTTCGTTC
ATCCATAGTTGCCTGACTCCCCGTCGTGTAGATAACTACGATACGGGAGGGCTTACCATCTGGCCCC
AGTGCTGCAATGATACCGCGAGACCCACGCTCACCGGCTCCAGATTTATCAGCAATAAACCAGCCAG
CCGGAAGGGCCGAGCGCAGAAGTGGTCCTGCAACTTTATCCGCCTCCATCCAGTCTATTAATTGTTG
CCGGGAAGCTAGAGTAAGTAGTTCGCCAGTTAATAGTTTGCGCAACGTTGTTGCCATTGCTACAGGC
ATCGTGGTGTCACGCTCGTCGTTTGGTATGGCTTCATTCAGCTCCGGTTCCCAACGATCAAGGCGAG
TTACATGATCCCCCATGTTGTGCAAAAAAGCGGTTAGCTCCTTCGGTCCTCCGATCGTTGTCAGAAG
TAAGTTGGCCGCAGTGTTATCACTCATGGTTATGGCAGCACTGCATAATTCTCTTACTGTCATGCCA
TCCGTAAGATGCTTTTCTGTGACTGGTGAGTACTCAACCAAGTCATTCTGAGAATAGTGTATGCGGC
GACCGAGTTGCTCTTGCCCGGCGTCAATACGGGATAATACCGCGCCACATAGCAGAACTTTAAAAGT
GCTCATCATTGGAAAACGTTCTTCGGGGCGAAAACTCTCAAGGATCTTACCGCTGTTGAGATCCAGT
TCGATGTAACCCACTCGTGCACCCAACTGATCTTCAGCATCTTTTACTTTCACCAGCGTTTCTGGGT
GAGCAAAAACAGGAAGGCAAAATGCCGCAAAAAAGGGAATAAGGGCGACACGGAAATGTTGAATACT
CATACTCTTCCTTTTTCAATATTATTGAAGCATTTATCAGGGTTATTGTCTCATGAGCGGATACATA
TTTGAATGTATTTAGAAAAATAAACAAATAGGGGTTCCGCGCACATTTCCCCGAAAAGTGCCACCTG
ACGTCTAAGAAACCATTATTATCATGACATTAACCTATAAAAATAGGCGTATCACGAGGCCCTTTCG
TCTTCAC
